# Case Report: Autoimmune Psychosis in Chromosome 22q11.2 Deletion Syndrome

**DOI:** 10.3389/fimmu.2021.708625

**Published:** 2021-10-14

**Authors:** Nicolás Lundahl Ciano-Petersen, Omar Hamad-Cueto, Hania Drissi-Reyes, Álvaro Doña-Díaz, Guillermina García-Martín

**Affiliations:** ^1^ Neuroimmunology and Neuroinflammation Group, Instituto de Investigación Biomédica de Málaga (IBIMA), Málaga, Spain; ^2^ Servicio de Neurología, Hospital Regional Universitario de Málaga, Málaga, Spain; ^3^ Andalucía Tech, Facultad de Medicina, Universidad de Málaga, Málaga, Spain; ^4^ Red Andaluza de Investigación Clínica y Traslacional en Neurología (Neuro-Reca), Málaga, Spain; ^5^ Servicio de Neurología, Hospital Clínico Universitario Virgen de la Victoria, Málaga, Spain; ^6^ Servicio de Urgencias, Hospital El Ángel, Málaga, Spain; ^7^ UGC Salud Mental, Hospital Universitario Virgen de la Victoria, Málaga, Spain

**Keywords:** chromosome 22q112 deletion syndrome, psychosis, autoimmune encephalitis, autoimmune psychosis, cognitive impairment

## Abstract

Chromosome 22q11.2 deletion syndrome (22q11DS) is characterized by congenital cardiac abnormalities, hypoplastic thymus, palatal abnormalities, and hypocalcemia, although other clinical features are frequent such as autoimmune and psychiatric disorders. One-third of the patients have psychotic disorders, frequently followed by developmental regression and long-term cognitive disturbances. Despite humoral and cellular immunodeficiency are common in 22q11DS, it is associated with an increased prevalence of autoimmune disorders such as idiopathic thrombocytopenic purpura and juvenile idiopathic arthritis, likely due to immune dysregulations associated with thymic abnormalities, which plays a major role in self-tolerance. We report an unique case of a 14-year-old girl with 22q11DS that presented with subacute psychotic symptoms, intolerance to antipsychotics, CSF pleocytosis, and EEG abnormalities, that was successfully treated with empiric immunotherapy after fulfilling criteria for probable seronegative autoimmune encephalitis and probable autoimmune psychosis. The autoimmune etiology of these clinical features of 22q11DS has never been postulated despite the predisposition of this syndrome to present autoimmune disorders. We suggest the systematic evaluation with serum and CSF neuronal antibodies, MRI, and EEG of patients with 22q11DS that develop subacute psychotic symptoms or rapidly progressive cognitive decline. Early immunomodulatory therapies should be carefully considered if criteria of probable autoimmune psychosis or possible autoimmune encephalitis are fulfilled, as it may prevent long-term disabilities. Further studies are required to assess the autoimmune origin of psychosis and cognitive impairment associated with 22q11DS.

## Introduction

Chromosome 22q11.2 deletion syndrome (22q11DS) is a genetic disease caused by a microdeletion of 3 million base pairs (Mb) on the chromosomal region 22q11.2 (1). Its prevalence is thought to be approximately 1:4000 births, although other studies estimate it to be higher (2). It is associated with multiple congenital malformations leading to heterogeneous clinical phenotypes (3), but cardiac anomalies, palatal abnormalities, hypoplastic thymus, and hypocalcemia are the most frequent reasons for obtaining genetic testing for 22q11.2 (1). Moreover, other clinical features are common such as psychiatric and immune disturbances (1).

One-third of the patients develop psychotic disorders during adolescence, such as schizophrenia, bipolar disorder, and psychotic depression (4). These conditions are similar to those of the general population, although psychosis in 22q11DS patients is frequently followed by long-term cognitive decline, and in some cases, seizures and neuroimaging volumetric changes (4). Cognitive and behavioral disturbances in 22q11DS have been associated with a higher risk for developing psychotic symptoms (4, 5), and patients with psychotic symptoms seem to develop a more intense cognitive decline (6), suggesting an underlying common origin of both features.

Immune disturbances are common in 22q11DS probably related to its association with thymic hypoplasia (3, 7). Recurrent infections are frequent due to cellular and humoral immunodeficiency during their life, especially when associated with low T-cell counts, the most commonly described immunologic feature in 22q11DS (7). Despite that they commonly present lymphopenia, they have a higher prevalence of autoimmune disorders such as idiopathic thrombocytopenic purpura and juvenile idiopathic arthritis (3, 7), likely due to the role of the thymus in T-cell selection and central self-tolerance (8). Thus, a higher prevalence of rare autoimmune disorders could be expected in these patients, such as autoimmune encephalitides (AE), which could be responsible for psychosis and cognitive impairment of some patients with 22q11DS.

In the last decades, the field of neuroimmunology has experienced a revolution due to the identification of neuronal antibodies responsible for a wide spectrum of AE, some with prominent psychiatric symptoms. A clinical diagnostic approach is possible, but a definite diagnosis is based on the presence of neuronal autoantibodies (9). However, solid evidence is suggesting that aberrant immunological responses driven by antibodies targeting neuronal antigens might contribute to psychiatric symptoms and cognitive impairment in certain subsets of patients. Therefore, the term autoimmune psychosis has been proposed for mild or forme fruste of AE with predominant psychotic symptoms. For these reasons, an alternative set of diagnostic clinical criteria have been proposed for patients with suspicion of an antibody-mediated AE with isolated or predominant psychotic symptoms (10), in order to ease its recognition and prompt early immunotherapy to achieve better long-term outcomes (11).

We present the case of a patient successfully treated with immunotherapy for a subacute psychotic disorder upon suspicion of AE, which subsequently led to the diagnosis of 22q11DS due to her previous cardiac history. Among other characteristic clinical features, 22q11DS patients frequently present with cognitive regression associated with predominant psychotic symptoms, which to our knowledge, have never been proposed to have an autoimmune origin. Since 22q11DS is associated with an increased prevalence of autoimmune disorders, the autoimmune origin of these symptoms should be included in the differential diagnosis of psychosis associated with 22q11DS.

## Case Description

We present the case of a 14-year-old woman with a previous history of Fallot Tetralogy and mild cognitive developmental delay that did not restrict her daily activities, that presented with subacute psychotic symptoms consistent on grandiose delusions, incoherent and incompressible speech with neologisms, and auditory hallucinations, in addition to insomnia and night terrors. In the previous month, her family noticed a progressive decline of her handwriting on homework, with lots of cross-outs and incomprehensible words, accompanied by inappropriate behavior with other family members. Initially, these psychotic symptoms were associated with her mild developmental delay and were treated with antipsychotics, such as haloperidol and risperidone. However, 5 days later her psychotic symptoms worsened, including irritability, disruptive and self-harming behaviors, accompanied by abnormal repetitive right hemicorporeal dyskinetic movements, eye-closing automatisms, and oculogyric crisis, which improved after discontinuation of these medications. Low doses of olanzapine were then introduced, achieving partial symptomatic control without extrapyramidal side effects. However, she presented a progressive decline in cognitive and motor skills, becoming bedridden in four weeks, requiring constant care for most daily activities, which equals a functionally score of 5 in the modified Rankin scale (mRS). This score is the most extended functional scale in neurology to assess the degree of disability/dependence of a patient. Although initially designed for stroke clinical trials, it is broadly used nowadays for assessing patients with AE (11).

**Figure 1:**
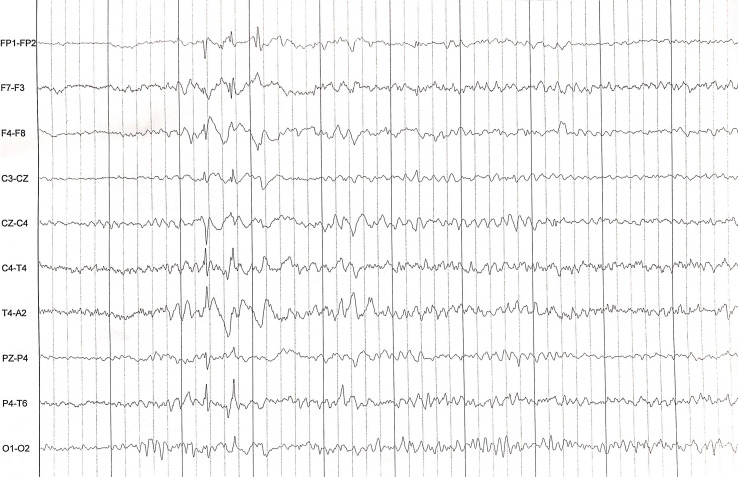
EEG showing bilateral frontoparietal polyspikes and spike-and-wave discharges, predominantly on the right hemisphere.

**Figure 2:**
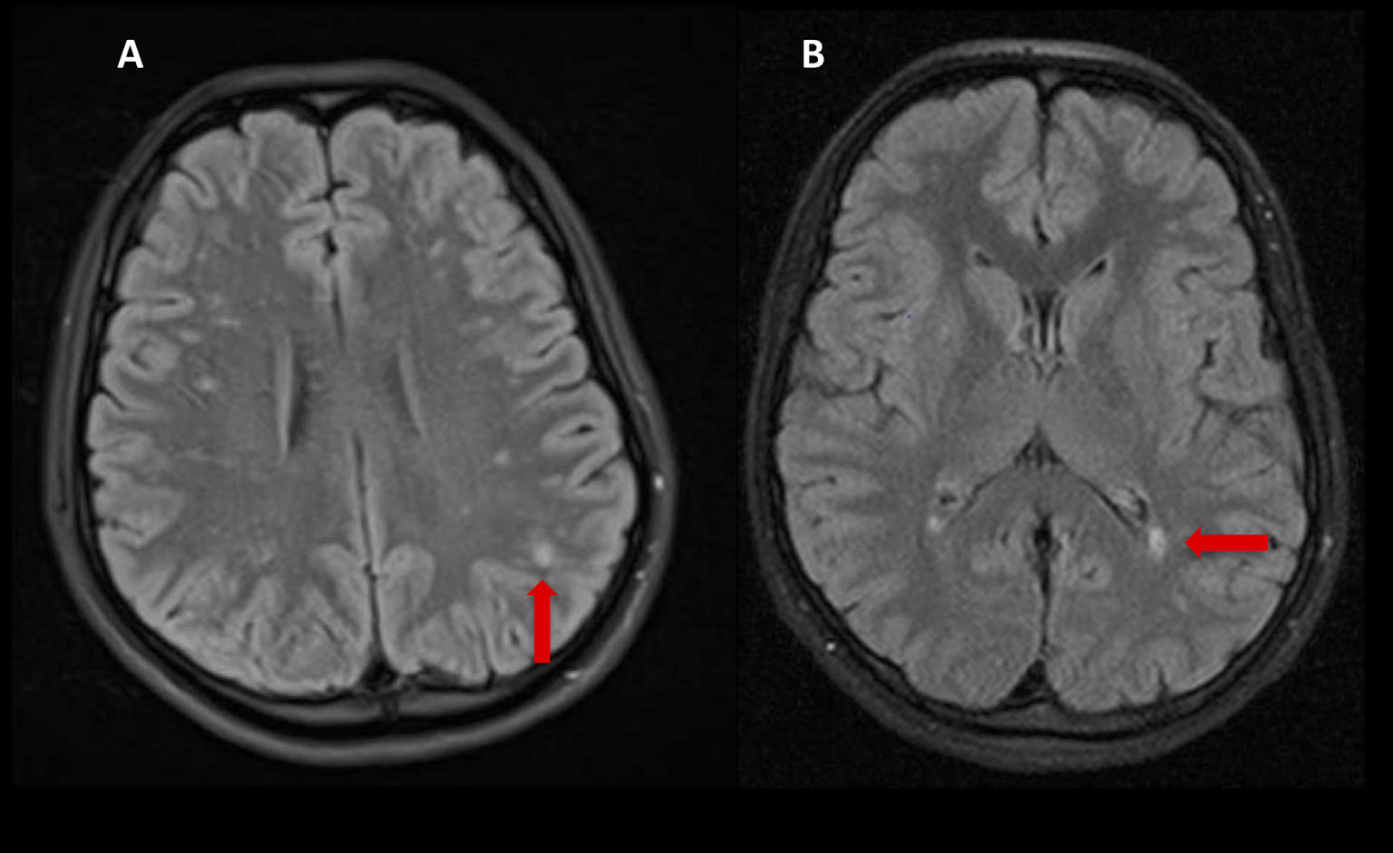
Brain MRI Flair sequences showing new confluent subcortical white matter lesions compatible with inflammation (red arrows) **(A, B)**.

**Figure 3:**
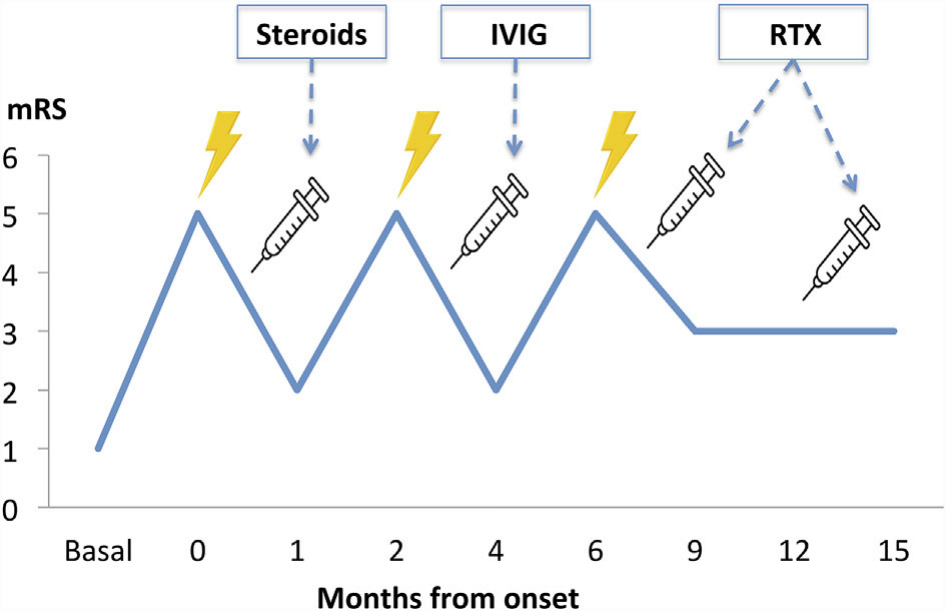
Clinical course timeline during the first 15 months of follow-up, with three clinical relapses and subsequent improvement after immunotherapy administration. IVIG, intravenous immunoglobulins; mRS, modified Rankin score; RTX, rituximab.

The rapidly progressive clinical course and antipsychotic intolerance raised the suspicion of AE and empirical intravenous 1g methylprednisolone was administered for 3 days in a different center, with an excellent response, especially regarding behavior and sleep quality. However, after steroids tapering, she presented a relapse and was transferred to a reference hospital. At this time, her physical and neurological examination was unremarkable, besides the aforementioned behavior disturbances. Brain MRI showed very few non-specific white matter lesions and electroencephalography (EEG) showed bilateral frontoparietal polyspikes and spike-and-wave discharges, predominantly on the right hemisphere (**Figure 1**). Cerebrospinal fluid (CSF) analysis showed 14 cells/μL (100% lymphocytes)(normal range = <5 cells/μL), 23.4 mg/dL proteins (normal range = <45mg/dL), normal IgG index and no oligoclonal bands. CSF viral polymerase chain reactions were negative. Serum and CSF neural surface antibodies including NMDAR, AMPA, GABAbR, LGI1, CASPR2, DPPX, and mGLUR5, and onconeural antibodies were negative. Routine analysis including ionized calcium, thyroid-stimulating hormone, infectious serology, autoimmune antibodies such as antinuclear antibodies, platelets, total lymphocyte count, and immunoglobulins subsets were unremarkable, and whole-body CT scan and 18F-fluorodeoxyglucose PET excluded a paraneoplastic origin. After reasonable exclusion of other alternative causes of subacute psychosis such as infectious encephalitis, drug toxicity, metabolic alterations, and other autoimmune disorders as systemic lupus erythematosus, a 5 days course of 0.4g/kg of intravenous immunoglobulin (IVIG) was administered, as she fulfilled criteria for probable autoimmune psychosis (10) and possible AE (9), supported by the previous good response to immunotherapy. At 6 months follow-up, she presented a new relapse, and full workup was repeated with unremarkable results, including neuronal autoantibodies. However, brain MRI showed new subcortical white matter lesions especially in the left parietal lobe (**Figure 2**), which considering the clinical scenario and the age of the patient were highly suggestive of AE, fulfilling the criteria for probable seronegative AE (9). Thus, she was started on rituximab (1g on days 1 and 15, and then 1g every 6 months) as a second-line treatment due to the disabling symptoms suggestive of refractory relapsing AE, with an excellent clinical response (**Figure 3**). After 24 months of follow-up, she had a good recovery reaching an mRS of 3, being able to have a partially autonomous life, attend school and maintain social relationships, despite cognitive disturbances being slightly worse than before the disease. She was maintained on lormetazepam and low doses of olanzapine, as well as on rituximab 1g every 6 months as an immunomodulatory treatment. In all follow-up analyses, her CD19+ B-lymphocytes were below 1%, and no adverse or unanticipated events were observed. Subsequent EEGs have not shown abnormalities and MRIs have been stable during the follow-up.

During the second work-up, a genetic analysis by Multiplex Ligation-dependent Probe Amplification was performed upon suspicion of 22q11DS because of her cardiac personal history and the new-onset autoimmune disorder, which found a microdeletion from CTCL1 to LZTR1 genes in chromosome 22q11.2. Before this diagnosis, no other finding had raised the suspicion of an immunodeficiency; immunoglobulins subsets, total lymphocytes count and lymphocytes subsets had always been in normal range, she had no previous personal or family history of recurrent infections or intellectual disability, and no serious adverse events had been reported after vaccination with live vaccines.

## Discussion

In this study, we describe a rare case of a 14-year-old patient with 22q11DS that presented a seronegative AE with prominent psychotic symptoms and a good response to immunotherapy. The autoimmune origin of psychotic and cognitive disabilities in patients with 22q11DS has never been proposed, regardless of the predisposition of these patients to develop autoimmune disorders.

The etiological diagnosis of psychotic disorders in young patients may be challenging, as autoimmune encephalitis may be misdiagnosed if other neurological features are not present (10). The clinical presentation of AE is extremely diverse and subacute psychotic symptoms may be the only clinical feature at onset, as AE associated with anti-N-methyl-D-aspartate receptor (NMDAR) antibodies (12). Although our main limitation is that neuronal antibodies were negative in our patient’s serum and CSF, which are mandatory to reach a definite diagnosis (9), she presented other characteristic clinical features that strongly suggest an NMDAR-like AE, such as paradoxical worsening of psychotic symptoms and dyskinetic movements after antipsychotic administration (12). Nevertheless, the psychotic symptoms could be explained by the inclusion of schizophrenia susceptibility genes as DGCR8, DGCR2, ZDHHC8, and PRODH within the microdeletion responsible for 22q11DS (13). Similarly, antipsychotic intolerance could be explained by the inclusion of the gene COMT, which encodes an enzyme involved in the dopaminergic metabolism (14). However, the autoimmune origin of these clinical findings is strongly supported by the presence of CSF pleocytosis, EEG abnormalities, the clinical and EEG response to immunotherapy, and subsequent relapses after its withdrawal.

In the last decade, many studies have focused on defining clinical red flags that should raise suspicion of an autoimmune origin of psychosis (9, 10), as early immunotherapy may have a great impact on the patient’s recovery (11). Among these clinical clues, the presence of a tumor, history of a recent cancer diagnosis, or previous history of immune disorders such as thyroid disease and systemic lupus erythematosus may support an immune origin of psychotic disorders (10). However, genetic disorders associated with increased autoimmunity have never been proposed as a clinical clue to consider an AE.

22q11DS is associated with recurrent infections due to humoral and/or cellular immune deficiencies (7), as well as with an increased risk of autoimmune disorders (1, 15), although the mechanisms underlying the latter are not fully understood. The self-tolerance breakdown responsible for this predisposition to autoimmune phenomena may be associated with the thymic abnormalities characteristic of 22q11DS (1), as it plays a major role in the selection and removal of T-cells reactive to self-antigens (8). Moreover, recent evidence points towards an imbalance in regulatory T-cells and Th17-cells being responsible for a proinflammatory environment in 22q11DS, mainly due to a severe reduction in regulatory T-cells and the anti-inflammatory IL-10, accompanied by high levels of IL-17, IL-6, and IL-23 (15). Additionally, recurrent infections associated with 22q11DS may also play a role in the pathogenesis of autoimmune disorders by molecular mimicry between viral and self-antigens, similarly to anti-NMDAR encephalitis after an herpes simplex virus encephalitis (16, 17).

In our case, no prodromal symptoms suggesting a post-infectious disorder were reported, and the diagnosis of 22q11DS was made retrospectively due to her previous history of Fallot syndrome. However, the clinical picture and the presence of CSF pleocytosis, EEG abnormalities, good clinical and EEG response to immunotherapy strongly supported the diagnosis of seronegative autoimmune encephalitis, in addition to the long-term stabilization despite most of these patients tend to present a cognitive decline after psychotic disturbances (4, 5). Since 22q11DS is associated with an increased risk of autoimmune disorders and a high prevalence of behavioral and cognitive disturbances (3, 7), a common autoimmune origin of the latter could be hypothesized, at least in some of these patients. Our results in a single case experience cannot be extrapolated to all 22q11DS patients, especially because we were not able to make a definitive diagnosis in absence of neuronal antibodies. However, clinicians should be aware of AE in patients with a personal history of 22q11DS that present rapidly progressive cognitive decline or subacute psychosis, especially if other common clinical features of AE are present, such as seizures or movement disorders. In these cases, a full workup including serum and CSF analysis for neuronal antibodies, brain MRI and EEG should be performed. If criteria for possible AE and probable autoimmune psychosis are fulfilled (9, 10), and other alternative causes have been reasonably excluded, immunomodulatory therapies could offer an alternative option to manage psychosis in 22q11DS. However, it should be carefully considered in patients with immunodeficiencies, as they are prone to infectious complications that could have devastating consequences. First-line therapies comprise steroids and intravenous immunoglobulins, which are overall safe and well-tolerated. On the contrary, second-line therapies including rituximab and cyclophosphamide are associated with a higher risk of adverse events, especially infectious complications; therefore, it should be restricted to severe cases with a definitive diagnosis of autoimmune psychosis or probable seronegative AE (9, 10).

Further studies are required to assess the possible autoimmune origin of psychosis and cognitive impairment in patients with 22q11DS since immunomodulatory therapies could prevent long-term disabilities in these patients. We suggest the systematic evaluation with the aforementioned ancillary testing of 22q11DS patients presenting with abrupt psychosis or rapidly progressive cognitive impairment, and if the criteria of probable autoimmune psychosis or possible autoimmune encephalitis are met, early empirical immunotherapy should be carefully considered.

## Data Availability Statement

The raw data supporting the conclusions of this article will be made available by the authors, without undue reservation.

## Ethics Statement

Written informed consent was obtained from the minors' legal guardian for the publication of any potentially identifiable images or data included in this article.

## Author Contributions

NC-P and GG-M: design and conceptualization of the study, major role in the acquisition and analysis of the data, and drafting the manuscript and revising for intellectual content. OH-C, HD-R, and ÁD-D: major role in the acquisition and analysis of the data, and revising the manuscript content. All authors contributed to the article and approved the submitted version.

## Conflict of Interest

The authors declare that the research was conducted in the absence of any commercial or financial relationships that could be construed as a potential conflict of interest.

## Publisher’s Note

All claims expressed in this article are solely those of the authors and do not necessarily represent those of their affiliated organizations, or those of the publisher, the editors and the reviewers. Any product that may be evaluated in this article, or claim that may be made by its manufacturer, is not guaranteed or endorsed by the publisher.
